# Use of a rapid test to assess plasma *Plasmodium falciparum* HRP2 and guide management of severe febrile illness

**DOI:** 10.1186/s12936-015-0900-3

**Published:** 2015-09-22

**Authors:** Ipsita Sinha, Nattwut Ekapirat, Arjen M. Dondorp, Charles J. Woodrow

**Affiliations:** Mahidol-Oxford Tropical Medicine Research Unit (MORU), Mahidol University, Bangkok, Thailand; Centre for Tropical Medicine and Global Health, University of Oxford, Oxford, UK

**Keywords:** Falciparum malaria, Severe febrile illness, PfHRP2, Plasma, Rapid test

## Abstract

**Background:**

Plasma *Plasmodium falciparum* histidine-rich protein-2 (PfHRP2) is the most accurate biomarker for severe malaria, but its measurement by ELISA has been considered too unwieldy to incorporate into clinical management.

**Methods:**

Plasma samples covering a wide range of PfHRP2 concentrations were applied to rapid diagnostic tests (RDTs). RDTs were read by eye and digital capture, and PfHRP2 concentrations were measured via serial dilution with results compared to ELISA readings.

**Results:**

The Paracheck^**®**^ brand showed the strongest correlation (r^2^ = 0.963) as well as the lowest inter-observer variability (combined kappa across band intensities for three observers = 0.938). Plasma PfHRP2 measurement via serial dilution showed minimal bias compared to ELISA and acceptable limits of agreement. Three different dilutions of a well characterized set of admission samples from uncomplicated and severe malaria patients studied in a low transmission setting gave an area under the receiver operator characteristic curve of 0.844 in terms of identifying severe malaria.

**Conclusions:**

These studies show that plasma PfHRP2 can be assessed via a single RDT, with application of a plasma dilution of 1:5 or 1:10 providing useful diagnostic information to assist in patient management or clinical trial inclusion.

## Background

In regions with medium to high intensity transmission of *Plasmodium falciparum*, diagnosing the cause of severe febrile illness is particularly challenging because of the high background prevalence of malaria parasitaemia. A malaria blood film or rapid diagnostic test (RDT) is often the only diagnostic available, but the presence of parasites is non-specific and malaria is frequently over-diagnosed, resulting in failure to consider and treat other life-threatening conditions [1]. Approximately a quarter of patients thought to have cerebral malaria in fact have other diagnoses on post-mortem [2].

Plasma *P. falciparum* histidine-rich protein-2 (PfHRP2), a soluble protein produced by sequestered parasites responsible for severe disease, provides a reliable method to distinguish patients with severe malaria from febrile patients with an alternative cause of severe illness. Plasma PfHRP2 concentrations define malaria severity and prognosis with substantially greater accuracy than parasitaemia in African children [3–5] and Papua New Guinean adults [6]. In African children with coma, plasma PfHRP2 concentrations accurately identified cerebral malaria defined by post-mortem findings [7] or malarial retinopathy [7–9]. In African children meeting criteria for severe malaria, but in the lower third of plasma PfHRP2 concentrations (<830 ng/ml), the mortality benefit of artesunate over quinine seen at higher PfHRP2 concentrations was entirely lost, a strong indication that death was due to other causes [10].

Identification of children with low plasma PfHRP2 levels is likely to benefit both individual patients and health systems, indicating the need for additional diagnostic procedures and treatments. Relevant investigations include cerebrospinal fluid examination in comatose children, blood cultures and imaging. Invasive bacterial infection is the most commonly identified alternative cause of severe febrile illness [11, 12] but blood culture, if available, has low sensitivity (particularly when there has been pre-treatment with antibiotics). Treatment of all severely ill patients with broad-spectrum antibiotics has been recommended [1] and is widely (but not completely) practised. However, deciding on the type and duration of antibiotics, and the benefit of adjunctive steroids, requires some knowledge of the likely cause and site of disease; furthermore tuberculous meningitis remains an important differential in febrile encephalopathy [13]. At a health system level, a knowledge of true incidence rates of severe malaria as well as the incidence of non-malarial diseases is important for guiding future diagnostic and treatment pathways in severe febrile illness, particularly as transmission levels fall. In addition, in areas of propagation of viral haemorrhagic fever, the decision to isolate at a relatively early stage of clinical management is likely to be influenced by knowing the malaria attributable risk. Point-of-care measurement of PfHRP2 is also relevant for enrollment in clinical trials and other prospective studies [14].

Measurement of plasma PfHRP2 has generally been undertaken via ELISA, taking several hours and requiring resources that are beyond the majority of laboratories in malaria endemic regions. Earlier work described the feasibility of using a standard diagnostic RDT to quantify plasma PfHRP2 [15]. Here these studies were extended by testing a number of RDT brands and quantifying the RDT readout both by eye and digital capture, comparing results to ELISA measurements and using an established reference sample for calibration. Relevant dilutions of plasma samples from severe and uncomplicated malaria patients in a low transmission setting were then tested to define the conditions under which useful assessment of plasma PfHRP2 in a high transmission setting might be achieved using a single RDT.

## Methods

### Samples

**Fig. 1:**
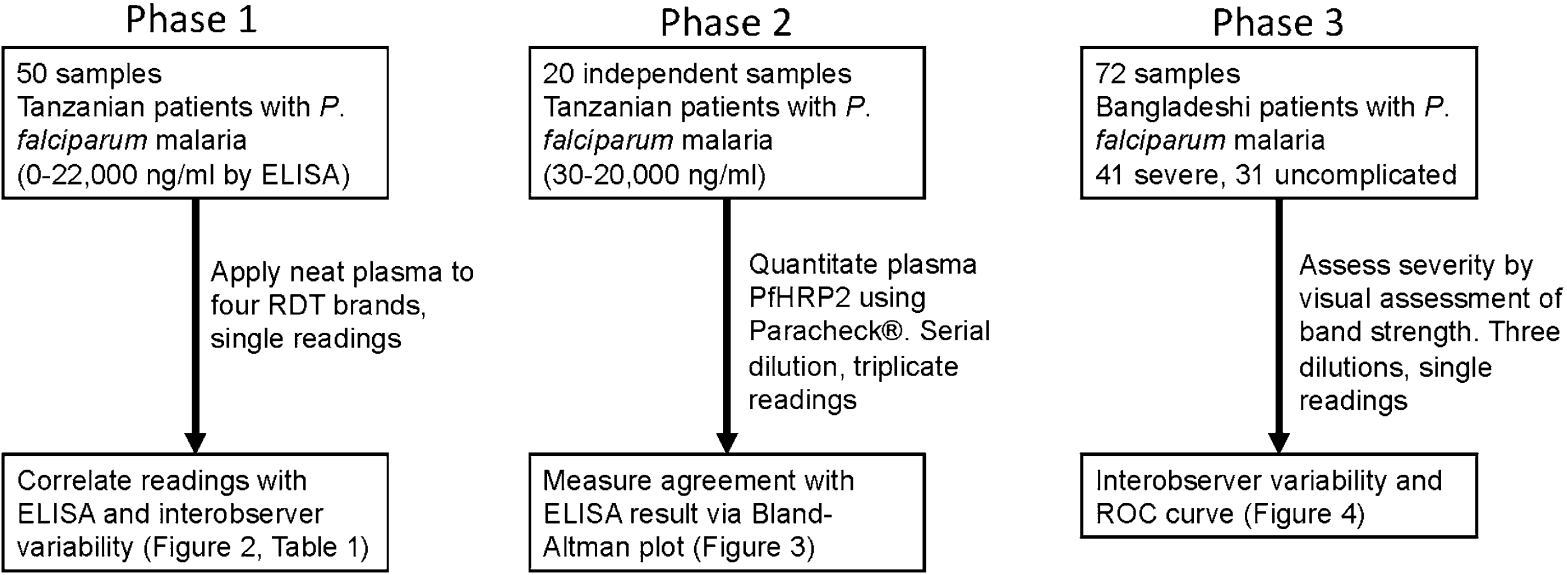
Workflow describing sample numbers and origins, kits and dilutions tested and outcomes analysed

Characterization of the conditions under which RDTs could be applied was undertaken in two phases with plasma samples from children recruited into the Teule, Tanzania site of the AQUAMAT severe malaria trial comparing quinine and artesunate [16] (Fig. 1). The performance of the RDT at individual sample dilutions to discriminate severe from uncomplicated malaria was then examined in plasma samples from non-pregnant adults (**≥**16-year-old) admitted to hospital with slide- or RDT-confirmed falciparum malaria and enrolled in prospective studies at Chittagong Medical College Hospital, Bangladesh (2011); uncomplicated and severe cases were defined using modified World Health Organization criteria [17]. ELISA methods and results and issues related to ethical permission for testing these samples have been previously described [10, 18].

### Choice of RDT

Four RDT kits solely based on PfHRP2 detection were studied: Core**™** Malaria Pf kit (MAL-190020, Core Diagnostics, UK), Paracheck^**®**^ Pf Device (V.3) (cat. 30301025, Orchid Biomedical Systems, India), First Response^**®**^ Malaria Antigen HRP2 kit (cat. I13FRC30, Premier Medical Corporation Ltd., India) and SD Bioline Malaria Ag Pf kit (cat. 05FK50, Standard Diagnostics, Korea). A single batch of each RDT was used for all work. All brands passed Phase 2 of Round 1–3 of the WHO testing programme [19].

### Reading of RDTs

The intensity of bands was assessed by digital scanning and visual assessment. Scanning was undertaken using a BioRad Gel Doc^TM^ XR+, using the Colorimetric application optimized for faint bands and capturing the maximum intensity of each selected area; on occasions small white spots just prior to the reaction line (areas that remain dry) rendered the mean value for the band an unreliable estimate of band intensity. As well as the PfHRP2 line itself, readings were taken from the background area of the nitrocellulose strip and the positive control band. For non-linear regression, this background intensity was subtracted from the test band result to produce a net reading representing the band strength relative to background.

After application of samples test kits were relabelled using a code and assessed by three independent observers blinded to the identity of each sample. Band intensity was graded as 0 (no visible band), 1 (faint line), 2 (faint band), 3 (clear band weaker than control) and 4 (clear band equivalent to or more intense than positive control).

Given that all tests were undertaken with plasma, it was appropriate to reassess the timeframe over which band strength remained stable visually and by digital scanning. In the first phase of work (testing of neat plasma samples on four kits, Fig. 1), visual readings were initially made between 20 and 40 min after the assay was performed, consistent with product recommendations when applying whole blood to the kits used. We soon observed that, stored in cool, dark, dry conditions, the strength of both control and test bands did not change over days or weeks, and digital scans were therefore considered to be accurate if taken within 1 month of the original assay. At the time of writing inspection of the test kits (which have been retained) confirms that visual band strength remains unchanged more than 2 years after the assays were undertaken.

### Statistics

Concentrations of plasma samples were determined by serial dilution and triplicate analyses, with the point of half-maximal intensity calculated by non-linear regression in GraphPad Prism with the lower asymptote set to zero after subtraction of background intensity. Agreement between visual assessment of concentration and ELISA readings was assessed by Bland–Altman analysis. Interobserver variability was based on the kappa-statistic.

## Results

### Dynamic range and selection of RDT for further work

**Fig. 2:**
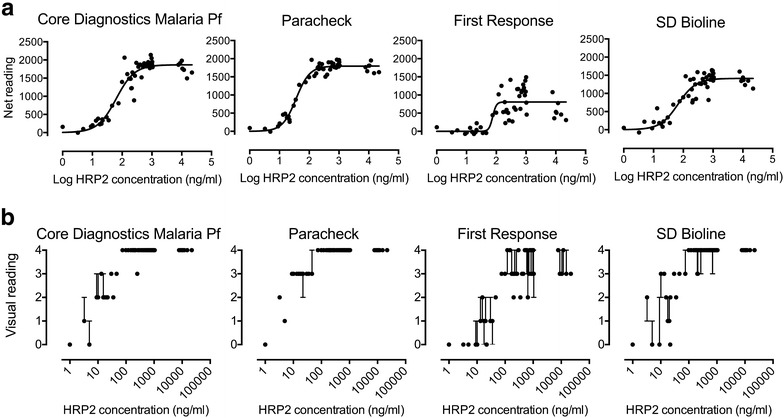
Comparison of four kits for assessment of plasma HRP2 level using 50 admission samples from the AQUAMAT study based on digital scanning (**a**) or visual assessment (**b**)

The work was undertaken in three phases (Fig. 1). Initial work using ten-fold dilutions of pooled malaria plasma obtained from severe malaria patients (at a concentration of 2500 ng/ml) showed that the dynamic range of the Paracheck^**®**^ RDT kit lay between 25 and 250 ng/ml PfHRP2 (data not shown). Fifty neat samples from Teule, Tanzania, with plasma HRP concentrations ranging from zero to 22,012 ng/ml were applied to each of four kits. The PfHRP2 concentration associated with half-maximal signal in each kit ranged from 36.2 (Paracheck^®^) to 75.9 ng/ml (First Response^®^) (Fig. 2; Table 1).

**Table 1:** Results of non-linear regression analysis of ELISA-measured plasma PfHRP2 concentration vs. digital reading, and of interobserver variability assessment for each of four kits

	Core™	Paracheck^®^	First Response^®^	SD Bioline
Digital assessment				
Top (reading units)	1867	1796	807.2	1414
HillSlope	1.408	1.904	7.058	1.453
EC50 (ng/ml)	63.14	36.25	75.91	66.99
EC50 (95 % CI)	48.38–82.40	31.69–41.48	49.63–116.1	47.35–94.80
Goodness of fit (r^2^)	0.9082	0.9627	0.5661	0.8516
Visual assessment kappa-statistic				
0—no visible band	0.7931	1	0.7785	0.7857
1—faint line	0.3197	1	0.3262	0.3056
2—faint band	0.7869	0.7432	0.6019	0.6296
3—clear band weaker than control	0.7778	0.9187	0.5879	0.6384
4—band at least as strong as control	1	0.9664	0.5662	0.85
Combined	0.8602	0.9377	0.5865	0.7198

The digital capture data showed that the strength of the band correlated well with the gold standard ELISA result for 3 of the 4 kits used, with the closest correlation found for the Paracheck^**®**^ kit (r^2^ = 0.9627; Fig. 2a). Given the similar dynamic ranges of the kits, it is unlikely that this was due to the range of concentrations studied, but rather the intrinsic properties of the kits.

Results from visual assessment closely agreed with scanning (Fig. 2b). Interobserver variability was lowest with the Paracheck^**®**^ (kappa-statistic for the combined categories = 0.938), indicating very good agreement across the three observers. Identification of strong test bands (category 4) or absent bands (category 0) from other bands appeared to be most robust, while defining bands of intermediate character (categories 1–3) was generally associated with poorer levels of agreement.

Seven samples with relatively high plasma concentrations (above 7000 ng/ml) were included in order to check for a possible prozone phenomenon, in which band intensity drops due to flooding of the test strip with free antigen. There was no convincing evidence of any reduction in signal in these highly concentrated samples for any of the kits.

### Quantitation of individual samples by dilution

**Fig. 3:**
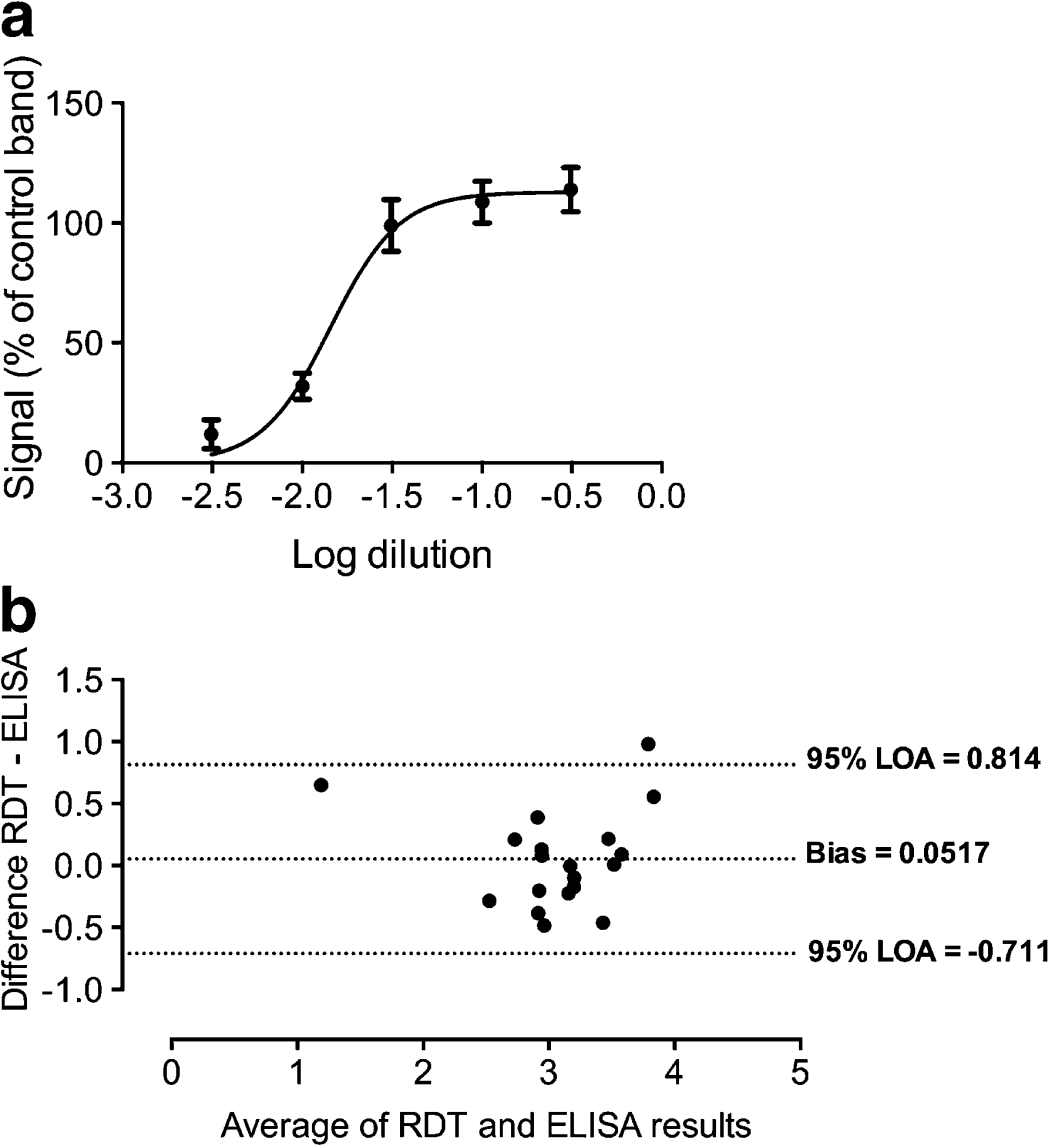
**a** Example dilution-signal plot from a serially diluted sample (tested in triplicate, with *error bars* indicating standard error of the mean). **b** Bland–Altman plot of RDT and ELISA-derived plasma PfHRP2 concentrations for clinical samples; data are log-transformed

The Paracheck^**®**^ kit was then used to test the ability of an RDT to quantitate plasma PfHRP2 in an independent set of 20 clinical samples. Samples underwent half-log serial dilutions in PBS producing 1:3.2, 1:10, 1:32, 1:100 and 1:320 dilutions. The neat sample was also applied if the 1:3.2 dilution failed to produce a strong band. These dilutions were applied in triplicate to Paracheck^**®**^ kits and digital readings obtained as previously (test band and background). A log dilution-signal curve was plotted and the EC50 (dilution required to produce a band half as intense as maximal intensity) calculated (Fig. 3a). By referencing to a pooled plasma control and using the ratio of the dilutions required to reach EC50, it was possible to calculate the absolute PfHRP2 concentration for each sample via RDTs only. A Bland–Altman plot using log-transformed data was used to examine agreement between the RDT-based method and ELISA. Overall the agreement was good, with no evidence of bias and limits of agreement around three-quarters of a log unit (approximately fivefold; Fig. 3b).

### Single RDT testing

**Fig. 4:**
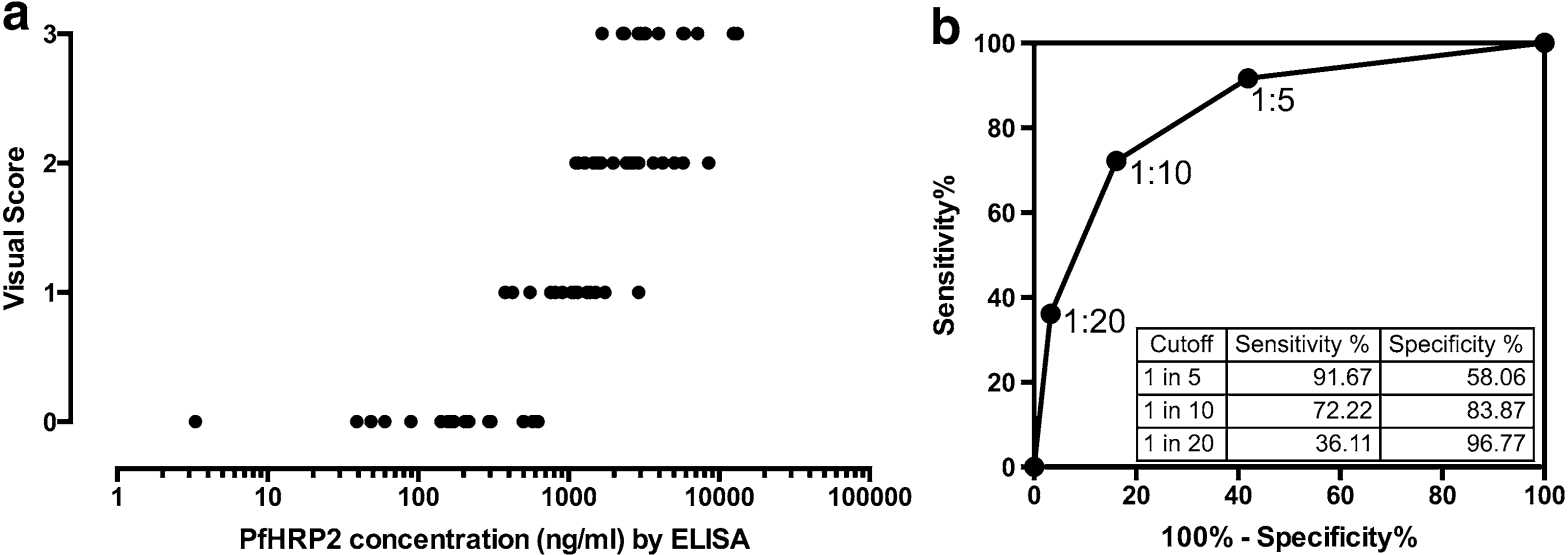
Relationship between concentration determined by ELISA and RDT based on three dilutions (**a**) and resulting ROC curve for identification of severe malaria (**b**). *Inset* to **b** the sensitivity and specificity based on each of the three sample dilutions are shown

The final step was to explore the accuracy of visual judgment using a single RDT to discriminate severe from uncomplicated malaria in 72 patients admitted with malaria in a low transmission setting (Chittagong, Bangladesh). Clinical severity assessment was considered as gold standard since incidental malaria in these areas is rare. The visually robust cut-off between a strong band (category 4) and lower strength bands (0–3) was used, with a band clearly weaker than positive control indicating a low probability of severe malaria. Choice of possible dilutions was informed by the mid-point of the dynamic range of the Paracheck^**®**^ RDT being found at approximately 50 ng/ml, with the cut-off between categories 3 and 4 somewhat higher. In previous work, suitable thresholds defining severe malaria (or lack of benefit of artesunate) were approximately 1000 ng/ml [7, 10], indicating that a dilution of approximately a log order of magnitude would be necessary to allow visual inspection to discriminate clinical state. Fixed dilutions of 1:5, 1:10 and 1:20 were tested. Three observers assessed the results, with the majority result taken for calculations of sensitivity and specificity. The kappa-statistic for assessment of strong compared to weaker bands was 0.8145 indicating good agreement. As expected, visual reading was able to pick out samples with lower PfHRP2 concentrations (Fig. 4a). Each sample was given a score according to the combined results from these three tested dilutions: 3, strong band at all three dilutions; 2, strong band at 1:10 and 1:5 only; 1, strong band at 1:5 only and 0, weak band at 1:5. Based on these scores the area under a receiver-operator characteristic (AUROC) curve for assessing disease state (defined by clinical criteria) was 0.844 (p < 0.0001) (Fig. 4b); the sensitivity and specificity for the three individual dilutions used are also shown in Fig. 4b.

## Discussion

There is still substantial room to refine the diagnosis of severe febrile illness in high transmission settings, and plasma PfHRP2 holds considerable promise in this regard. No other biomarker for cerebral malaria has demonstrated the high positive and negative predictive values seen for plasma PfHRP2 [20], and it is already the basis of most RDTs used for diagnosis of uncomplicated *P. falciparum* malaria. A PfHRP2-based RDT to define plasma levels would allow practical assessment in low-resource settings and could be the first field-based test since microscopy to provide information on the contribution of malaria in severe ill children [20]. The work reported here demonstrates conditions under which a single malaria RDT can provide semi-quantitative information on plasma PfHRP2 levels and hence identify African children in whom there is a low probability that severe malaria is the cause. Several PfHRP2-based RDTs hold potential to be applied to this task, although among the brands studied here, the Paracheck^**®**^ RDT appeared to have the tightest correlation with ELISA as well as close inter-observer agreement. The measured agreement also appeared superior to previous studies of dipstick assessment for this purpose [15].

The area under the receiver operator characteristic curve for a single RDT in discriminating severe from uncomplicated malaria was found to be 0.844, based on admission plasma samples from an established cohort of uncomplicated and severe adult malaria patients studied in a low transmission setting [18] (Bangladesh). In these patients malaria was unlikely to be an incidental cause of illness; indeed any cases in which severe illness was primarily due to another analysis would have led to an underestimation of performance. Levels of plasma PfHRP2 in the uncomplicated and severe groups appear to be comparable to those from children in high transmission settings [7, 18]. However testing on plasma from children in high transmissions settings, whose level of severity of malaria is established either by post-mortem or retinal examination [7], would provide a more definitive measurement of accuracy.

This work used a single batch of the Paracheck^**®**^ RDT for all work. Batch-to-batch variation is a well known issue with RDTs, and in long-term studies calibration to a reference sample would be indicated, along the same lines reported for ELISA methodology [10].

Practical implementation of the test currently requires prior separation of plasma from whole blood and, since all the RDTs studied showed maximal signal well below a threshold relevant to discriminating severe disease, dilution of plasma before application to the RDT. The ideal plasma dilution needs to be linked to the expected proportion of cases that the test is targeted to identify, in this case apparent cases of severe malaria in which the true cause of illness is another disease. For example, in a group of one hundred severely ill patients where this proportion is one quarter (derived from the post-mortem studies of Taylor et al. [2]), the presence of weak band at a 1:10 plasma dilution would identify 21 of the 25 target patients in whom the cause of severe illness is not malaria, but also mistakenly flag 21 of 75 true severe malaria cases. A 1:5 dilution would pick up 15 of the target population of 25, while incorrectly flagging only 6 of the severe malaria cases. A lower dilution would hence be more appropriate for a setting with relatively lower malaria transmission.

## Conclusions

In summary, this work shows that a single RDT can be used to obtain useful information on plasma HRP2 concentration and hence the underlying cause of severe febrile illness. Application in the field would be enhanced by engineering a simpler testing approach, for example by development of an RDT in which plasma selectively enters the nitrocellulose strip at an appropriate concentration. Incorporation into management algorithms holds promise to refine the diagnosis of severe febrile illness, a common syndrome associated with high mortality.
